# Airway and Swallowing Outcomes in Children With Combined Laryngomalacia and Laryngeal Cleft

**DOI:** 10.7759/cureus.58914

**Published:** 2024-04-24

**Authors:** Caleb Escudero, Zachary Tanenbaum, Rahal Riad, Rebecca Weiner, Sohit P Kanotra

**Affiliations:** 1 Otolaryngology - Head and Neck Surgery, University of Iowa Hospitals and Clinics, Iowa City, USA; 2 Pediatric Gastroenterology, University of Iowa Stead Family Children's Hospital, Iowa City, USA; 3 Pediatric Medicine, University of Iowa Stead Family Children's Hospital, Iowa City, USA; 4 Otolaryngology - Head and Neck Surgery, University of California Los Angeles, Los Angeles, USA

**Keywords:** pediatric, dysphagia, aspiration, deep notch, laryngeal cleft, laryngomalacia

## Abstract

Laryngomalacia (LM) and laryngeal cleft (LC) can independently cause dysphagia but rarely can occur concomitantly. We discuss the presentation, decision-making, and swallow outcomes following surgical correction of combined LM and LC. We present four patients with combined LM and an LC who underwent both primary supraglottoplasty (SGP) and laryngeal cleft repair (LCR). Each patient presented with recurrent choking or coughing with feeds. Stridor was only present in two patients. Patients with SGP saw the resolution of stridor when present, but dysphagia persisted in all four cases. LCR clinically and objectively resolved all symptoms of dysphagia. We found that flexible fiberoptic laryngoscopy is not always reliable at detecting combined pathology. Patients presenting with persistent dysphagia following SGP should be suspected of having interarytenoid pathology. We recommend a staged surgical approach with SGP before LCR.

## Introduction

Pediatric dysphagia is a common, yet challenging problem that can be life-threatening. Approximately 1% of all children will experience dysphagia [1,2], with this number being even larger for high-risk populations such as premature and developmentally delayed children [2,3]. Additionally, pediatric dysphagia can be multifactorial, which can further complicate diagnosis and management [2]. Both laryngomalacia (LM) and laryngeal cleft (LC) are known to cause dysphagia independently [2,4,5], but rarely they can both present concomitantly.

LM is characterized by the collapse of the supraglottic airway. It is the most common cause of pediatric stridor and is associated with respiratory and feeding problems, which can cause failure to thrive, present in approximately 9% of patients [4,6,7]. Approximately 49% to 75% of patients with LM have at least one abnormal swallowing study, regardless of their subjective symptoms of dysphagia [4,8]. In cases of severe LM, surgical management may be required in the form of a supraglottoplasty (SGP). Patients with LM undergoing an SGP may be expected to have transient dysphagia postoperatively, even in the absence of preoperative dysphagia [9]. However, dysphagia improves over time with 75% of nonsyndromic patients having complete resolution of dysphagia by 14 months, as compared to 44% in syndromic patients [9]. 

An LC is a congenital disorder in which the posterior laryngotracheal wall does not completely close, allowing solids or liquids to pass from the esophagus into the trachea, resulting in aspiration [5,10-13]. LCs are graded in severity from type 1 to 4 based on the depth of the defect [14]. It has been reported that type 1 LCs (LC-1) are present in up to 7% of pediatric patients requiring laryngoscopic evaluation for dysphagia [10]. LC-1, the least severe form, can present with recurrent choking episodes, a chronic cough, and/or recurrent upper respiratory infections [5,10-12]. Although injection laryngoplasty has previously been used to manage LC-1s, a recent meta-analysis showed better clinical outcomes with primary surgical repair [10]. Endoscopic management has been shown to resolve dysphagia in approximately 71% of patients with LC-1 [10]. A deep interarytenoid notch (DIN) is a variant of LCs in which the interarytenoid height is less than 3 mm but does not meet the criteria of a complete LC [14]. There is currently little research surrounding DIN outcomes, and current guidelines indicate that LC-1s and DINs should be managed the same way [14].

The scenario can be increasingly complicated in children with combined LM and LC-1/DIN. Due to the rarity of combined pathology, many questions remain unanswered including the decision-making regarding surgical correction of each pathology, expected swallowing outcomes, and expected clinical benefit. While the release of the aryepiglottic folds can be performed after a laryngeal cleft repair (LCR) to alleviate the narrowing of the airway, the present study focuses on cases presenting with dysphagia in the setting of both LM and LC. The purpose of this case series is to discuss our decision-making process and to investigate the swallowing outcomes in this rare subset of patients. This article was previously presented as an oral presentation at the 2023 SENTAC Annual Meeting on December 12, 2023.

## Case presentation

We present four cases of patients requiring both SGP and LCR as primary procedures. While the release of the aryepiglottic folds can be performed after an LCR to alleviate the narrowing of the airway, the present study focuses on cases presenting with dysphagia in the setting of both LM and LC. Both surgeries were performed under sedation without intubation. In each SGP, the primary surgeon preferred the use of a flexible fiber CO_2_ laser, which was used to ablate redundant arytenoid mucosa. To surgically manage LC-1s/DINs, endoscopic LCRs were performed using the bulk closure technique after lasering the interarytenoid region with a flexible fiber CO_2_ laser set to ultrapulse mode. Under microscopic visualization, the defect was lasered on medial and lateral edges and reapproximated with 3-0 or 4-0 vicryl sutures. Postoperative ventilation and tracheostomy were not required in all four cases.

Case 1

Patient 1 presented as a 23-month-old male with a history of gastroesophageal reflux disease (GERD) and recurrent respiratory tract infections for which he followed with gastroenterology and pulmonology. He was evaluated by otolaryngology after he began to have recurrent coughing fits with feeds. Before this, the patient had been reaching developmental milestones appropriately and was neurologically intact. These coughing episodes occurred at least once per meal and were accompanied by recurrent vomiting. A videofluoroscopic swallow study (VFSS) showed mild pharyngeal dysphagia with delayed swallow reflex, decreased laryngeal elevation, and delayed airway closure, resulting in deep penetration of thin liquids. Flexible fiberoptic laryngoscopy revealed a deep interarytenoid notch, suggestive of LC. The patient was placed on thickened liquids initially, but failed conservative treatment and was taken to the OR for direct laryngoscopy. This revealed the patient to have heavily redundant arytenoid tissue consistent with LM that was obstructing the airway and a 2 mm interarytenoid notch. At this time, an SGP with CO_2_ laser ablation of redundant tissue was opted to be completed initially, with staged LCR to occur at a later date should symptoms not resolve. The patient tolerated surgery without complications. Two months following the SGP, the patient’s airway symptoms had improved significantly, but his dysphagia remained subjectively and on repeat VFSS. As dysphagia symptoms were not resolved, the patient was taken back to the OR for an LCR. The patient tolerated surgery without complications. One month postoperatively from the LCR, the patient was no longer having choking or coughing episodes with feeds. A swallow study demonstrated a mildly abnormal pharyngeal stage, but the patient was within functional limits and there was no concern for aspiration with a normal diet. At one year follow-up, the patient had no further issues and had recovered fully.

Case 2

Patient 2 was a male born at 25 weeks gestation via c-section with a history of retinopathy of prematurity, patent foramen ovale, bronchopulmonary dysplasia, and chronic feeding difficulty who presented at seven months old (chronological age) to otolaryngology due to history of aspiration. The patient was neurologically intact, but he required an NG tube for most of his feeds and 2L O2 via nasal cannula. At the time of presentation, he was underweight in the 0.13 percentile. Several swallow studies showed aspiration with thin liquids, but not with nectar-consistent liquids. In office flexible fiberoptic laryngoscopy showed type 1 LM. The patient failed conservative management and at his follow-up appointment one month later, a functional endoscopic evaluation of swallowing (FEES) showed evidence of a deep notch. The patient had a G-tube placed while awaiting his OR date with otolaryngology. At 13 months old, he underwent direct laryngoscopy, revealing a 2 mm interarytenoid notch and type 1 laryngomalacia. SGP was performed at the time of direct laryngoscopy. The patient tolerated surgery without complications.

**Figure 1:**
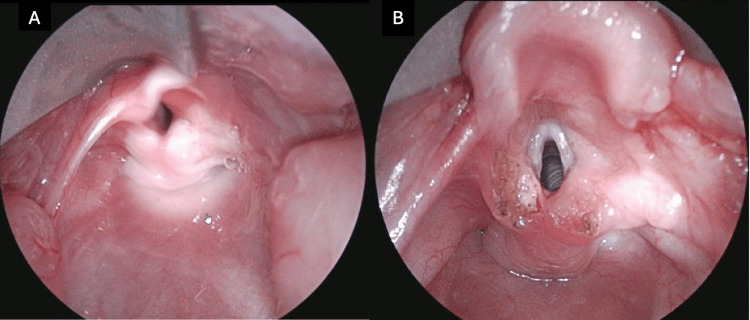
Patient 2's larynx (A) preoperatively and (B) post-supraglottoplasty. Post-laryngeal cleft repair images are unavailable.

One month postoperatively, the patient was still reliant on G-tube feeds and continued to demonstrate aspiration with liquids. His swallow studies showed persistent aspiration at three months postoperatively, and he was taken to the OR for an LCR. The patient tolerated surgery without complications. Two months after his LCR, the patient was improving clinically and was able to tolerate thickened feeds orally. However, he continued to have aspiration on swallow study. The patient was able to tolerate spoon feeds without thickeners four months postoperatively, passed a swallow study without evidence of aspiration or penetration, and his weight increased to the second percentile. He was eventually able to discontinue G-tube feeds at six months postoperatively and has tolerated oral intake without issue (Figure 1).

Case 3

Patient 3 was a female born at 37 weeks gestation and was noted to be small for gestational age with poor feeding. Before her presentation, the patient had been reaching developmental milestones appropriately and was neurologically intact. She presented to otolaryngology at 20 days old with intermittent stridor, reflux, tracheal tugging, intercostal retractions, coughing, and perioral cyanosis. She had multiple urgent care and emergency room visits and was admitted following observation of desaturation to 83% and significant coughing with feeds. Otolaryngology found the aryepiglottic folds to be tight with redundant arytenoid mucosa with hooding anteriorly, suggesting the presence of congenital LM. A swallow study demonstrated mild pharyngeal dysphagia with impaired laryngeal closure, resulting in mild aspiration attributed to LM. The patient was taken to the operating room (OR) for direct laryngoscopy where a diagnosis of type 2 LM was made with subsequent SGP improving it to type 1. The patient tolerated surgery without complications. The patient’s stridor appeared to resolve with SGP. Swallow study at two days postoperatively showed a slight improvement in airway protection, and eventually, the patient was discharged. 

Four days after discharge, the patient began to experience recurrent coughing spells, desaturations, and a single cyanotic episode with feeds. She was evaluated three days later, and her feeding appeared to be improving. Over the next six months, the patient developed worsening nighttime coughing, post-tussive emesis, and recurrent respiratory infections that required albuterol and nebulizer treatments, leading to referral to the multidisciplinary aerodigestive clinic. 

Upon evaluation in the aerodigestive clinic, a swallow study indicated pharyngeal swallow delay with decreased laryngeal elevation, resulting in consistent deep penetration of thin liquids and decreased strength at the tongue base. Triple endoscopy revealed regression to a grade 2 subglottic stenosis with significant lymphoid tissue hypertrophy. Clinical history was suspicious for LC, which was confirmed in the OR on direct laryngoscopy. A 3 mm intra-arytenoid notch was found and closed. The patient tolerated surgery without complications.

**Figure 2:**
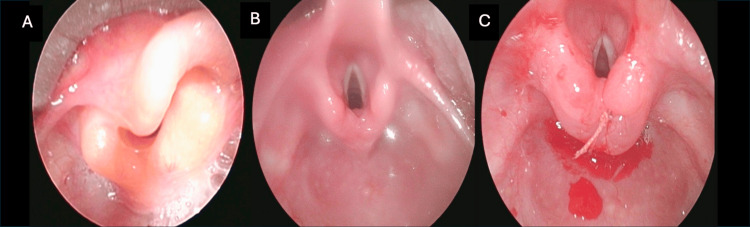
Patient 3's larynx (A) preoperatively, (B) post-supraglottoplasty, and (C) post-laryngeal cleft repair.

One month later, the patient had improved significantly. There were no episodes of choking, aspiration, choking, or cough. A VFSS demonstrated a mildly abnormal pharyngeal stage and very mild transient penetration with large-volume drinks. However, the patient was within functional limits and was capable of eating a regular diet with no modifications. At her most recent two-year follow-up, the patient was completely asymptomatic (Figure 2).

Case 4 

**Figure 3:**
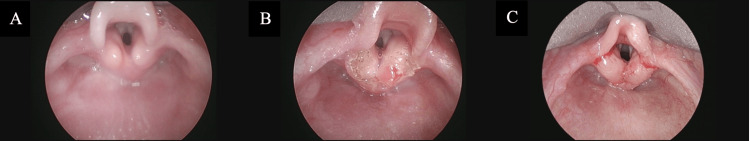
Patient 4's larynx (A) preoperatively, (B) post-supraglottoplasty, and (C) post-laryngeal cleft repair.

Patient 4 presented as a 48-day-old previously healthy male with increased work of breathing, stridor, cyanosis, and choking/coughing with feeds. Before this, the patient had been reaching developmental milestones appropriately and was neurologically intact. Flexible fiberoptic laryngoscopy showed redundant arytenoid tissue consistent with type 1 LM. Swallow study at this time demonstrated penetration into the laryngeal vestibule. He was taken to the OR for direct laryngoscopy, which showed type 1 LM and a deep interarytenoid notch, and an SGP was performed. The patient tolerated surgery without complications. One month postoperatively, the patient’s stridor and desaturations had improved, but choking with feeds persisted. FEES did not reveal any aspiration. The patient failed conservative therapy of thickened feeds, Famotidine, and Flonase. Five months postoperatively the patient's swallow study demonstrated moderate-to-severe pharyngeal dysphagia with aspiration of liquids. An NG tube was placed while awaiting to be taken back to the OR for an LCR. Due to social issues, the patient was unable to return to the OR until he was 11 months old. At this time, he was taken for an LCR for his DIN. The patient tolerated surgery without complications. At two months postoperatively, the patient showed significant improvement. He was no longer coughing or choking with liquids or solids. Swallow study at this time showed a single episode of silent aspiration, and the patient was placed on thickeners. At four months postoperatively, the patient had no further concerns clinically and the swallow study showed no evidence of aspiration. The patient has been advancing his diet as tolerated and has not exhibited any signs of recurrence (Figure 3).

In summary, dysphagia was the primary complaint in each case, and only two children were stertorous. Three of the patients were dependent on tube feeds before intervention. VFSS demonstrated aspiration preoperatively and following SGPs. LCRs resolved each patient’s subjective and objective dysphagia. Each patient tolerated surgery without complication. Each case is summarized in Table 1.

**Table 1:** Demographic information, patient presentation, weights perioperatively, and surgical findings. DIN, deep interarytenoid notch; GERD, gastroesophageal reflux disease; BPD, bronchopulmonary dysplasia; LC-1, type 1 laryngeal cleft; AE, aryepiglottic; LCR, laryngeal cleft repair

Patient #	Sex	Age at presentation (months)	Age at SGP (months)	Age at LCR (months)	Weight at presentation (kg)	Weight after SGP (kg)	Weight after LC repair (kg)	Presenting symptoms	Flexible fiberoptic findings	Surgical findings	Mode of nutrition	Comorbid conditions	
													1	M	23	25	27	12.6	13	13.2	Dysphagia, stridor, cough, choking, recurrent respiratory infections, emesis, exercise-induced laryngomalacia	Normal	Laryngomalacia with complete airway obstruction, 2 mm DIN	PO	GERD and asthma	
2	M	8	10	13	6.7	7.17	8.5	Failure to thrive, aspiration, dysphagia, recurrent respiratory infections, BPD	Redundant mucosa over arytenoids	Type 1 laryngomalacia, 3 mm DIN	G tube	Prematurity, global developmental delay, bronchopulmonary dysplasia, patent foramen ovale	
3	F	1	1	6	10.7	11.7	12.6	Feeding difficulties, coughing, perioral cyanosis, croup, recurrent respiratory infections	Redundant mucosa over arytenoids	Adenoid hypertrophy, laryngomalacia, 2 mm LC-1	NG tube	GERD and asthma	
4	M	1	1	11	5.2	5.7	9.4	Choking, coughing, stridor, increased work of breathing	Redundant mucosa over arytenoids, shortened AE folds	Type 1 laryngomalacia, 2 mm LC-1	NG tube	None	

## Discussion

In this series, we present four cases in which both SGP and LCR were performed in the setting of combined LM and LC-1/DIN. These were the only four patients treated by the primary surgeon who were found to have combined pathology. Due to the lack of research surrounding combined pathology, this series attempts to describe patient presentation and swallowing outcomes following staged surgical correction, as well as raise other important questions that need to be answered in future studies. 

In our experience, combined pathology presented in all four patients as swallowing dysfunction characterized by choking/coughing with feeds and a chronic wet cough. Stridor was only present in two out of four cases despite the presence of LM. Pulmonary presentations were also common in three out of four patients, with recurrent respiratory infections retrospectively attributed to recurrent aspiration. These patients were all neurologically intact, and despite patients often being underweight, they were reaching developmental milestones. Even with thorough preoperative assessment, diagnosis of multifactorial dysphagia is difficult, as in-office laryngoscopy and swallow studies are not always reliable in the detection of combined pathology. Preoperative swallow studies in each patient showed consistent aspiration or penetration, which may be attributed to either LM or LC-1/DIN [4,5,8,10-13]. In-office laryngoscopy demonstrated LM in each patient, but LC-1/DINs were not detected in two out of four patients. Due to these challenges, LM may hide the presence of LC-1/DIN. In each case, a swallow study following SGP showed persistent dysphagia in all patients. This can be transiently expected, but dysphagia should not persist [4,8]. Therefore, patients presenting with persistent dysphagia following SGP should be assessed for LC-1/DIN.

Even when combined pathology is detected, it can be a diagnostic dilemma when symptomatic patients are found to have LM and a deep notch, as both pathologies are potential sources of dysphagia. While LM can resolve over time as the patient grows, we felt operative management was required as these patients had poor growth as a result of their dysphagia. In these cases, we feel that a staged surgical approach is beneficial, as it helps to identify the primary pathology related to the patient’s symptoms. In each of our cases, we first proceeded with SGP. This choice was made because LCR leads to the narrowing of the laryngeal inlet and often necessitates the release of the folds via secondary SGP [15,16]. Therefore, performing SGP first may prevent additional surgical procedures in patients whose airways are already widened or whose dysphagia is the result of LM. SGP was performed with a laser used in ultra-pulse mode, which has been shown to decrease surgical suspension time [17]. In this subset of cases, we performed LCR at a later date if the postoperative swallow study continued to show persistent aspiration after SGP. We avoided a single-stage repair of SGP and LCR to prevent the production of an extensive area of raw tissue, which predisposes the patient to supraglottic stenosis. Following LCR, three out of four patients showed no signs of aspiration six weeks postoperatively. A single patient required an additional two months of observation before seeing resolution. Overall, patients appeared to have improved stridor and ceased to have recurrent respiratory infections following SGP but required LCR for resolution of their dysphagia.

The study has some limitations. This study only examined patients from a single surgeon and, due to the rarity of combined pathology, there were a limited number of cases. Additionally, due to the strategy of a staged repair, it is not completely clear if SGP can be an effective treatment for combined pathology on its own. There may be other cases in which LM is the primary disorder with the presence of silent LC-1/DIN, but aspiration resolved following SGP, thus not requiring LCR and not meeting inclusion criteria. However, in these cases, LC-1/DIN is likely not severe enough to be a primary cause of the patient’s symptoms in the first place. Future studies should examine surgical strategies, including single versus staged repair, as well as the order in which SGP and LCR are performed.

## Conclusions

Combined pathology of LM and LC-1/DIN represents a challenge from a diagnostic and management standpoint. Patients presenting with persistent dysphagia following SGP should be suspected of having an LC-1/DIN and should undergo surgical closure of the defect when present. When both pathologies are detected, we feel that a staged approach to management is beneficial, as it helps to determine the primary source of dysphagia and avoids creating a large area prone to subglottic stenosis. We suggest that SGP should be performed before LCR in cases of combined pathology because this strategy potentially reduces the number of surgical procedures performed. 
